# Diversity, Phylogeny, anticancer and antimicrobial potential of fungal endophytes associated with *Monarda citriodora* L.

**DOI:** 10.1186/s12866-017-0961-2

**Published:** 2017-03-07

**Authors:** Meenu Katoch, Shipra Phull, Shagun Vaid, Shashank Singh

**Affiliations:** 1 Microbial Biotechnology Division, Indian Institute of Integrative Medicine, Jammu, India; 2 Cancer Pharmacology Divison, Indian Institute of Integrative Medicine, Jammu, India

**Keywords:** Endophytic fungi, *Monarda citriodora*, Antimicrobial activity, Tube dilution method, Cytotoxicity, MTT assay

## Abstract

**Background:**

Present study focuses on diversity and distribution analysis of endophytic fungi associated with different tissues of the *Monarda citriodora* Cerv. ex Lag. (Lamiaceae/Labiatae). Anticancer and antimicrobial potential of isolated endophytes have also been investigated.

**Results:**

A total of twenty eight fungal endophytes belonging to 11 different genera were isolated from this plant. All the endophytic fungi belonged to the Ascomycota phylum. The leaves were immensely rich in fungal species, while roots showed the highest tissue specific fungal dominance. Out of 28 fungal species, 72% endophytic extracts were found cytotoxic against one or more human cancer cell lines. The most prominent anticancer activity (IC_50_ value <10 μg/mL) was shown by MC-14 L (*Fusarium oxysporum*), MC-14 F (*F. oxysporum*), MC-18 L (*Aspergillus fumigatus*), MC-24 L (*Cladosporium tenuissimum*), MC-25 L (*Fusarium* sp.), MC-26 F (*F. oxysporum*) extracts. 75% of the extracts showed antimicrobial activities in agar disc-diffusion assay and 27% in the tube dilution method (MIC <100 μg/mL) respectively against the tested pathogens. Extracts of MC-14 L (*F. oxysporum*) and MC-18 L (*A. fumigatus*) displayed broad spectrum antimicrobial activity.

**Conclusions:**

These results indicated that *M. citriodora* harbors a rich fungal endophytic community with anticancer and antimicrobial activities. The isolated endophyte MC-24 L (*C. tenuissimum*) has the potential to be a source of novel cytotoxic/antimicrobial compounds. This is the first report of diversity of fungal endophytes isolated from *M. citriodora*.

**Electronic supplementary material:**

The online version of this article (doi:10.1186/s12866-017-0961-2) contains supplementary material, which is available to authorized users.

## Background

Endophytes are one of the most important parts of plant microbiota. These microbes reside inside the plants in variety of relationship but they do not cause any deleterious effect to them [1]. Endophytes are ubiquitous in nature and are found in every plant. They coexist inside the plant and are beneficial for better adaptability of the plants in their ecological niche [1–3]. During this process, endophytes interact with each other and their host plant changing the metabolic ability of the plant. In addition endophytes are known to produce a multitude of secondary metabolites of pharmaceutical importance [4, 5]. These metabolites belong to diverse structural classes and show variety of bioactivities including antifungal, antibacterial, antiviral, anticancer, antidiabetic, insecticidal, immunosuppressive, antioxidant etc. [6–9]. Some of the natural products such as cephalosporins, lovastatin, ivermectin, rapamycin, cyclosporine, paclitaxel etc. still find use as immunosuppressant and to control the infectious and parasitic diseases, lipid disorders, cancer and hypertension [10, 11].

Increasing incidence of cancer and development of superbug’s due to antibiotic resistance are the major challenges for researchers [12, 13]. Therefore, search for safer and novel drug based on natural product is of utmost importance. The success can be achieved by selecting the underexplored and/or unexplored biological resources. They are expected to be the producer of new chemical entities. Endophytic fungi belonging to unique environment are one of the promising sources of novel and natural drugs [10, 14].

*Monarda* commonly named as horsemint, beebalm or wild bergamot belongs to Lamiaceae (Labiatae), the mint family. It is vastly distributed, medicinal, aromatic and ornamental herb which grows annually or perennially [15, 16]. USA, Canada, and Mexico are its origin point but it also occurs in Europe and Asia [15, 17]. In India, it is growing in Shivalik hills since 1990. It consists of roughly thirty species and is being grown as garden, food and/or medicinal plant [18]. It is used as a flavoring agent in drinks, bakery and meat products. Its leaves were pulverized and sprinkled on meat to repel insects and to preserve the meat. Its decoction is used to cure catarrh, colds, toothaches, headaches, gastric disorders, nausea, menstrual pain, insomnia, to lessen fevers, soothe sore throat and to relieve flatulence. It is topically used for curing skin eruptions and infections. Its aroma is used in potpourri. Traditionally it has been used to treat variety of respiratory, digestive and skin disorders. Its use as febrifuge, diaphoretic, antirheumatic, carminative, sedative, diuretic and stimulant has also been reported [16]. The plant also has antiseptic, anti-oxidant and antifungal properties [19–22]. Major component of its essential oil, thymol, is now-a-days used in modern commercial mouthwash formulations [20, 22, 23]. Recently its essential oil is found to have anticancer property targeting PI3K pathway [24].

The economic importance of *M. citriodora* L. created an enthusiasm to study the endophytic community associated with this plant and their spatial distribution. They were also explored for antibacterial and anticancer activities. This attempt is novel as endophytes from *M. citriodora* have not been documented.

## Methods

### Collection, identification and authentication of plant material

Fully matured *M. citriodora* plants were collected randomly between March – April, 2013 from Shivalik hills, Jammu and Kashmir (32.73°N 74.87°E, altitude of about 1073 ft), India. The species was identified by taxonomist via leaf and flower morphology and preserved in the herbarium (accession no. 18554) and in the farm of IIIM as genetic resource.

### Isolation of endophytes

The endophytic fungi were isolated from *M. citriodora* as described by Strobel and Daisy [1] with slight modifications. Different tissues (leaves, roots and flowers) of the disease free plants were carefully excised with a sterile scalpel. In first instance, these tissues were cleaned by thorough washing in running tap water, followed by deionized (DI) water. Clean tissue pieces were sterilized in series of solution: 70% ethanol; 1% sodium hypochlorite (v/v); again 70% ethanol for 1 min each. Finally they were again rinsed with sterile distilled water two times. After surface sterilization, tissues were dried on blotting sheets and cut into 1 cm square pieces. These sterile small pieces were placed on water and potato dextrose agar (PDA) plates containing streptomycin (250 μg mL^−1^) to inhibit the bacterial growth. At the same time, water used for washing the tissues (100 μL) was also plated on the PDA to confirm the effectiveness of surface sterilization. The plates were incubated at 25 ± 2 °C after wrapping with parafilm and observed daily. The fungal mycelia which started growing from the tissues were subcultured on new PDA plates. The obtained endophytic fungal isolates were coded according to their tissue of origin (MC-1 L, MC-2 L, MC-3 L, etc. from leaves, MC-13R, MC-20R, from roots and MC-7 F, MC-14 F, MC-21 F, from flowers)*.* These *e*ndophytes were stored in paraffin oil at 4 °C and were deposited in RN Chopra, Microbial Repository, IIIM.

## Characterization of endophytes

### Morphology based identification of endophytes

The endophytic fungi were characterized on the basis of the morphology observed during their growth on PDA. They were also examined microscopically to determine the structure of hyphae, conidia, conidiophores and their arrangement.

### Molecular identification

Fungal endophytic isolates were finally identified by ITS based rDNA sequencing. Genomic DNA of the endophytes was extracted from the in vitro grown biomass of endophytes using the protocol described by Reader and Broda [25]. Approximately 1 g of dried mycelia was kept in liquid nitrogen and crushed to a fine powder. It was transferred to 10 mL extraction buffer and vortexed thoroughly. The samples were incubated in water bath set at 65 °C for 30 min followed by intermittent mixing. The tubes were centrifuged at 10000 *g* for 5-10 min followed by extraction with chloroform:isoamyl alcohol (24:1). Aqueous layer was collected and DNA was precipitated with 2.5–3 volume of absolute ethanol in presence of 1/10^th^ volume of sodium acetate (3 M pH 5.2). Tubes were inverted slowly to mix the contents followed by centrifugation at 8000 *g* for 20 min at 4 °C. Consequently, white/transparent pellets were washed with ice cold 70% ethanol followed by air drying. Dried pellets were dissolved in 20 μL of water (molecular biology grade). ITS sequences containing ITS1-5.8S-ITS2 spanning 500-600 bp were amplified with the universal primers ITS1 (5’-GGAAGTAAAAGTCGTAACAAGG-3’) and ITS2 (3’-TCCTCCGC TTATTGATATGC-5’) [26]. PCR reaction was set up in 50 μL containing DNA (1–10 ng), 1× PCR buffer (with 15 mM MgCl_2_), each dNTP (200 mM), each primer (10 pmol, Sigma, USA) and 1U *Taq* DNA polymerase (Bangalore Genei, India). Cycling parameters were 5 min at 94 °C followed by 30 cycles of 94 °C for 30 s, 55 °C for 1 min, 72 °C for 1 min and a final extension for 10 min at 72 °C. The PCR product (10 μL) was resolved using agarose gel electrophoresis at 100 V. The amplified product was purified using a Gel extraction Kit (Qiagen, USA) and sequencing reaction was set up in a 10 μL: 40–60 ng of purified PCR product, 3.2 pmol forward/reverse primer, Big Dye Terminator sequencing mix 8 μL (v. 3.1, Applied Biosystems). Samples were sequenced on an automated sequencing system (Applied Biosystems). Resultant sequences (KU527781-KU527806, KU680345, KU680346) were submitted to a Genbank and were blasted against the nucleotide database using blastn Tool of the US National Centre for Biotechnology Information (NCBI) for final identification of endophytes [27].

### Phylogenetic evaluation of endophytic fungi

For phylogenetic evaluation, endophytic ITS DNA sequences of present study and downloaded sequences of their nearest neighbors were aligned in Alignment Explorer of MEGA4 software [28] using ClustalW option. Trimming and verification of the sequence alignment were carried out using the MUSCLE (UPGMA) algorithm [29]. The Maximum Composite Likelihood and Neighbor-Joining methods were used to compute the evolutionary distances and history respectively [30, 31]. The robustness of the tree was assessed by bootstrap analysis with 1000 replication [32].

### Fungal diversity evaluation

To evaluate and quantify the endophytic fungal diversity associated with *M. citriodora*, different indices such as Menhinick’s index *(D*_*mn*_ *)*, Camargo’s index (1/*D*_*mn*_) or species richness, Fisher’s log series index (α), Simpson’s index (*D*), Simpson’s diversity index (1- *D*), the Shannon diversity index (*H’*), and Margalef’s richness (*D*_*mg*_) were calculated [33–36]. The similarity indices for fungal endophytic assemblages among tested tissues were also evaluated using Sorensen’s index (QS), and Jaccard’s index using the following equation: [QS = 2*a*/(2*a* + *b* + *c*)]; [JS = *a/*(*a* + *b* + *c*)] respectively, where *a* means the number of common fungal species found in endophytic populations of two different tissues and *b* and *c* mean the number of endophytic fungal species specific to tissues under comparison [37].

## Bioactivity evaluation

### Fermentation and extraction

For the extraction of molecules, the endophytic fungal isolates (twenty eight) were cultured on a set of five potato dextrose agar plates for a period of 15 days at 25 ± 2 °C in an incubator (New Brunswick, USA). 5 mm mycelial plug of 10-day old culture was used as inoculum. After fifteen days, fungal growth of five petri dishes was homogenized thoroughly with 12.5 mL of methanol. Homogenate was extracted with one volume of methylene chloride (DCM) (HPLC grade). The extraction process was repeated four times. Solvent containing extract was striped off in a rotary evaporator. The stock solutions of extracts (10 mg mL^−1^) were prepared in dimethyl sulfoxide (DMSO) and were used to evaluate the anticancer and antimicrobial potential.

### Cytotoxic activity

Endophytic extracts were tested for their cytotoxic effects using an MTT assay. It is a colorimetric assay to quantify the cell survival and proliferation. Four human cancer cell lines: HCT-116 (colorectal carcinoma), A-549 (lung), MCF-7 (breast), PC-3 (prostate) were procured from National Centre for Cell Sciences (NCCS), Pune, India for the present study. The MTT assay was performed as described by Pathania et al. [24]. Cells were cultured in RPMI-1640 medium with 10% fetal calf serum (FCS), and 100U penicillin/100 μg mL^−1^ streptomycin. Cells were incubated at 37 °C with 98% humidity and 5% CO_2_ environment (in CO_2_ incubator, Thermo Electron Corporation, USA). The cell density was adjusted to 10^5^ cells mL^−1^ of RPMI medium and 200 μL of these cells were plated in a 96-well plate. Fungal extracts in different concentrations (10–100 μg mL^−1^) were added to these wells and were incubated for 48 h. After 48 h, the medium of the wells was replaced with a medium containing 3-(4, 5-dimethylthiazol-2-yl)-2, 5-diphenyltetrazolium bromide (MTT, 100 μg mL^−1^) for additional 3 h. Subsequently, supernatant was aspirated out and 200 μL DMSO was used to dissolve the MTT-formazan crystals. An ELISA reader (Thermo Labs, USA) was used to measure the optical density of 96 well plate at 540 nm with 620 nm as reference wavelength. 5-FU, paclitaxel, and adriamycin drugs were used as positive controls while DMSO was used as negative control. Extracts were also evaluated on normal cell line (FR2) in parallel. Absorbance of treated versus untreated cells was recorded and percent growth inhibition was calculated. CurveFit software was used to calculate IC_50_ value.

### Antimicrobial activity

The extracts prepared from the endophytes were evaluated for antimicrobial activity. For antimicrobial activity, six test bacteria [*Bacillus subtilis* (MTCC No. 121), *Pseudomonas aeruginosa* (MTCC No. 424), *Salmonella typhimurium* (MTCC No. 98), *Escherichia coli* (MTCC No. 118), *Klebsiella pneumoniae* (MTCC No. 109), and *Staphylococcus aureus* (MTCC No. 737)] and yeast [*Candida albicans* (MTCC No. 183)] were purchased from Microbial Type Culture Collection (MTCC).

### Agar disc-diffusion assay

The endophytic extracts were tested for their antimicrobial and antifungal activity using agar disc-diffusion method [38, 39]. We used streptomycin and amphotericin B as positive controls while DMSO (0.5%) was kept as negative control. Each of the bacterial strain was grown in nutrient broth at 37 °C with 200 rpm for 16 h in an incubator. Cell density of the culture was adjusted to the McFarland standard turbidity (0.5), which is equivalent to 1.5 × 10^8^ colony forming units (CFU) mL^−1^ [38]. It was further diluted to give approximately 10^5^ CFU mL^−1^. For antibacterial assay, Mueller Hinton agar plates (90 mm, containing 20 mL medium) were spreaded with 100 μL of these cells. Stock solutions of fungal extracts and antibiotics were prepared to a conc. of 10 mg mL^−1^ and 1 mg mL^−1^ respectively. Fungal extracts (20 μL) and antibiotics (5 μL) were applied on sterile discs (6 mm diameter) in the centre of plates. All plates were incubated overnight at 37 °C. For antifungal activity, yeast extract peptone dextrose agar was used. Zone of inhibition (ZI) in terms of the diameter of area showing no bacterial growth around the disc was recorded in mm. The assays were repeated thrice and the mean value of three replications was calculated.

### Tube dilution method

For quantitative antimicrobial and antifungal activity, endophytic extracts were evaluated by tube dilution method [39]. Different dilutions of fungal extracts (12.5–100 μg mL^−1^) and antibiotics (6.5–50 μg mL^−1^) were prepared from the stock solutions. For each pathogen, 100 μL of 10^4^ CFU mL^−1^ was mixed with 900 μL of each dilution of fungal extracts and antibiotics. Three replicates of each sample were processed. Appropriate positive, negative and blank controls (virgin media) were also prepared in triplicate. All tubes were incubated overnight at 37 °C. The lowest inhibitory/ bactericidal concentrations (MIC/MBC) were recorded. The assays were replicated thrice and the mean values were calculated.

### Statistical analysis

All the collected parameters were examined with ANOVA and TUKEYS post hoc analysis using Graph Pad Prism software.

## Results

### Identification and characterization of the endophytic fungi

Endophytic fungi were isolated from healthy and symptomless tissues (leaves, roots and flowers) of *M. citriodora* to access their diversity, phylogeny and bioactive potential. Twenty eight endophytes were isolated. Absence of any growth on PDA, plated with water obtained from last rinse of tissues suggests that efficient surface sterilization has been performed. Endophytes were identified by their colony morphology and microscopic examination (Additional file 1: Figure S1 Table S1). Further their molecular identification was carried out by ITS based rDNA sequence analysis. Details of the fungal endophytes, their isolation source, GenBank accession numbers, and closest sequence homolog are given in Table 1.

**Table 1:** Summary of the fungal endophytes isolated from various tissues of *M. citriodora*

S No.	Endophyte*	EMBL-Bank accession number	Most closely related strain (accession number)	Maximum Identity (%)
1	MC-1 L	KU527781	*Colletotrichum boninense_* JQ676184	100
2	MC-2 L	KU527782	*Fusarium chlamydosporum*_KP641161	99
3	MC-3 L	KU527783	*Curvularia aeria* _KP131939	99
4	MC-4 L	KU527784	*Alternaria alternata*_GQ121322	100
5	MC-5 L	KU527785	*Aspergillus flavus*_KM285408	99
6	MC-6 L	KU527786	*Neosartorya hiratsukae*_GQ461906	98
7	MC-8 L	KU680345	*Aspergillus* oryzae_KT964480	93
8	MC-9 L	KU527787	*Penicillium commune*_KF938402	99
9	MC-10 L	KU527788	*Muscodor yucatanensis*_KJ572191	99
10	MC-12 L	KU527789	*Fusarium solani* _FJ426390	99
11	MC-14 L	KU527790	Fusarium oxysporum_JX406507	99
12	MC-15 L	KU527791	*Aspergillus* sp*.*_GQ352493	99
13	MC-16 L	KU527792	*Neurospora* sp._KJ676544	99
14	MC-17 L	KU527793	*Aspergillus waksmanii_*EF669934	99
15	MC-18 L	KU527794	*Aspergillus fumigatus*_KM207771	99
16	MC-20 L	KU680346	*Cladosporium* sp*.*KP050606	89
17	MC-24 L	KU527795	*Cladosporium tenuissimum*_KJ589554	100
18	MC-25 L	KU527796	*Fusarium* sp*._*KC007281	100
19	MC-13R	KU527797	*Alternaria carthami* _JF710542	99
20	MC-20R	KU527798	*Cladosporium* sp*._*JQ388271	99
21	MC-7 F	KU527799	*Fusarium* sp*.*_KJ567458	99
22	MC-14 F	KU527800	*Fusarium oxysporum*_KT876658	99
23	MC-17 F	KU527801	*Colletotrichum gloeosporioides_*KM520010	99
24	MC-21 F	KU527802	*Cladosporium cladosporioides*_ KP900248	99
25	MC-22 F	KU527803	*Fusarium oxysporum_* KF264963	100
26	MC-23 F	KU527804	*Gibberella intermedia_*JQ846048	99
27	MC-25 F	KU527805	*Fusarium redolens_*KJ540090	99
28	MC-26 F	KU527806	*Fusarium oxysporum_* KF998987	99

*last alphabet in the endophyte code represents the plant part to which endophyte was isolated i.e. L- leaf, R- Root and F- Flower etc

The isolated endophytic fungi belonged to 11 different genera. The maximum numbers of endophytes (18 isolates) were hosted by leaves followed by flowers (8 isolates) and finally roots (2 isolates) (Table 1). All the endophytic fungi belonged to the Ascomycota phylum. Out of these, 46.5% isolates belonged to class Sordariomycetes followed by Eurotiomycetes (28.5%), and Dothideomycetes (25%). The Sordariomycetes were represented by the orders *Hypocreales, Xylariales, Glomerellales,* and *Sordariales* with most of them being distributed in leaves and flowers. Eurotiomycetes were isolated from leaves alone and were represented by the order *Eurotiales,* whereas Dothideomycetes were isolated from all tissues and were represented by the order *Pleosporales* and *Capnodiales. Fusarium* spp., *Aspergillus* spp., and *Cladosporium* spp. were dominant over other species. In leaves, *Aspergillus* spp. (27%) occurred with the highest frequency followed by *Fusarium* spp. (22%), *Cladosporium* spp. (11%) while *Fusarium* spp. (62.5%) was dominant in flowers. The *Fusarium* spp. were found associated with both leaves and flowers*,* where as *Cladosporium* sp. was found associated with leaves and roots. Endophytes specifically isolated from the leaves tissues were *Curvularia aeria, Neosartorya hiratsukae, Muscodor yucatanensis, Neurospora* sp*., Colletotrichum boninense, Alternaria alternata, Penicillium commune, Fusarium* spp*., Aspergillus* spp*.,* and *Cladosporium* spp. whereas *Gibberella intermedia, Fusarium* spp*., Cladosporium cladosporioides*, and *Colletotrichum gloeosporioides* were found specific to flowers. Roots harbor only *Alternaria carthami* and *Cladosporium* sp*.*

### Phylogenetic evaluation

**Fig. 1:**
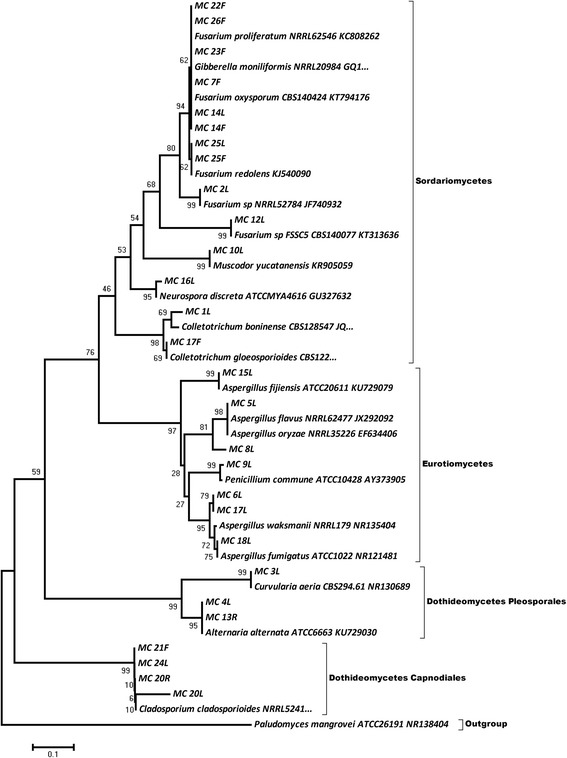
Phylogenetic position of the endophytic fungal isolates obtained from different tissues of the *M. citriodora.* The evolutionary history was inferred using neighbor-joining method [30]*.* The percentage of replicate trees in which the associated taxa clustered together in the bootstrap test (1000 replicates) is shown next to branches [32]. The evolutionary distances were computed using the maximum composite likelihood method [31]. Phylogenetic analyses were conducted in MEGA4 [28]

The phylogenetic tree describes the taxonomic relationship between the fungal endophytic species recovered from various tissues of *M. citriodora* (Fig. 1). In the phylogenetic tree, the sequences were clustered into four groups representing Sordariomycetes, Eurotiomycetes, and Dothideomycetes classes. Sequences belonging to order *Pleosporales* and *Capnodiales* were clustered separately. *Paludomyces mangrovei* ATCC26191 was laid down separately as out group. In the bootstrap analysis with 1000 replicates, bootstrap values (the percentage of clustering the associated taxa together) were computed and shown next to the branches in the phylogenetic tree indicating the stability of tree/subtree.

### Fungal diversity analysis

The diversity of the endophytic fungal populations isolated from different tissues was evaluated by various indices such as Fisher alpha diversity index (*α*), Shannon index *H*, Simpson’s diversity index (*1-D*), Margalef richness index (*D*_*mg*_) (Table 2). The species richness (*D*_*mn*_) was highest in the leaves (4.24) followed by the flowers (2.82) and roots (1.41). The tissue-specific fungal dominance was the highest in the roots (0.71), followed by flowers (0.47) and leaves (0.24). The most dominant species isolated from the leaves and flowers was *Fusarium oxysporum* with a relative proportion *P*_*i*_ (0.14) followed by *Fusarium* sp. (*P*_*i*_ *=* 0.07), whereas the dominant species isolated from the leaves and roots was *Cladosporium* sp. with a relative proportion *P*_*i*_ (0.07). The rest of the species were less dominant (*P*_*i*_ *=* 0.03). The Shannon, Simpson’s (0.94) and Margalef indices respectively revealed a high certainty and consistency of endophytic fungal species in the roots (0.69) and highly diverse and taxonomically rich fungal endophytes in leaves. Species evenness was uniform in leaves and roots, whereas slightly lower (0.88) in flowers. Global beta diversity indices were recorded as follows: Whittaker diversity index: 1.7692, Harrison diversity index: 0.076923, Cody diversity index: 4, Routledge: diversity index 0.2969, Wilson-Shmida diversity index 3.6923, Mourelle diversity index: 0.16054, Harrison diversity index 2: 0.021739, Williams’s diversity index: 0.33333. Sorenson’s and Jaccard’s (Jc) similarity indices were mentioned in Table 3. These indices indicate the significant diversity of endophytes within and between the different tissues of *M. citriodora*.

**Table 2:** Tissue specific diversity indices of endophytic fungi isolated from *Monarda citriodora*

Indices	Leaves	Roots	Flowers
Taxa_S	18	2	6
Individuals	18	2	8
Simpson’s Dominance_D	0.055	0.5	0.218
Simpson_1-D	0.944	0.5	0.781
Shannon_H	2.890	0.693	1.667
Evenness_e^H/S	1	1	0.883
Brillouin	2.022	0.346	1.102
Menhinick richness index	4.243	1.414	2.121
Margalef richness index	5.882	1.443	2.404
Equitability_J	1	1	0.930
Fisher_alpha diversity index	0	0	10.91
Berger-Parker dominance	0.055	0.5	0.375
Chao-1	171	3	16

**Table 3:** Jaccard’s (Jc) and Sorenson’s similarity indices for endophytic fungi of *Monarda citriodora*

Jaccard’s index/ Sorenson’s index	Leaf	root	Flower
Leaf	1.0	0.1	0.16
Root	0.05	1.0	0.0
Flower	0.09	0.0	1.0

## Bioactivity evaluation

### Anticancer activity

Anticancer activities of the endophytic extracts at the 100 μg mL^−1^ concentration were found against four human cell lines (HCT-116, MCF-7, PC-3, and A-549) while they didn’t inhibit the growth of normal cells (Table 4). Nearly 72% of the extracts showed cytotoxic activity against one or the other human cancer cell line with percent growth inhibition ranging from 51–100%. In the present study, sensitivity of cell lines against tested extracts was in the following order: PC-3 > MCF-7 > HCT-116 > A-549 cell lines. Thirty nine percent of the extracts showed >50% growth inhibition on all the tested human cancer cell lines.

**Table 4:** Anticancer activity of the extracts (100 μg mL^−1^) prepared from endophytic fungi isolated from *M. citriodora*

	Tissue		Lung	Colon	Breast	Prostate
S.No.	Cell line type		A549	HCT116	MCF7	PC-3
	Code	Conc (μg mL^−1^)	Percent Growth inhibition			
1	MC-1 L	100	13 ± 0.8	0 ± 0	13 ± 0.3	16 ± 0.5
2	MC-2 L	100	35 ± 1	22 ± 0.3	52 ± 0	10 ± 0
3	MC-3 L	100	33 ± 0.5	12 ± 0.4	20 ± 0.4	41 ± 0.4
4	MC-4 L***	100	67 ± 1	65 ± 0.2	91 ± 0.2	79 ± 0.3
5	MC-5 L	100	0 ± 0	47 ± 0	76 ± 0	51 ± 0.4
6	MC-6 L	100	27 ± 0.5	35 ± 0.5	54 ± 0.5	56 ± 0.4
7	MC-8 L***	100	98 ± 1	99 ± 0.4	94 ± 0.4	95 ± 0.4
8	MC-9 L	100	22 ± 0.8	10 ± 0.2	14 ± 0.3	48 ± 0.2
9	MC-10 L	100	47 ± 0	18 ± 0.4	45 ± 0.4	49 ± 0
10	MC-12 L	100	34 ± 1	29 ± 0.6	37 ± 0.2	58 ± 0
11	MC-14 L***	100	96 ± 1	100 ± 0.2	94 ± 0.3	100 ± 0.2
12	MC-15 L	100	24 ± 0.8	51 ± 0.8	86 ± 0	59 ± 0.5
13	MC-16 L***	100	59 ± 0	47 ± 0.2	88 ± 0.2	61 ± 0.2
14	MC-17 L	100	48 ± 1	23 ± 0	23 ± 0.5	25 ± 0
15	MC-18 L***	100	100 ± 0	93 ± 0.5	100 ± 0.2	90 ± 0.2
16	MC-20 L***	100	77 ± 1	82 ± 0.5	77 ± 0.2	63 ± 0.5
17	MC-24 L***	100	77 ± 1	99 ± 0.4	88 ± 0.4	96 ± 0
18	MC-25 L***	100	88 ± 1	100 ± 0.2	94 ± 0.3	99 ± 0.4
19	MC-13R	100	40 ± 1	20 ± 0.6	35 ± 0.2	50 ± 0.5
20	MC-20R	100	10 ± 1	1 ± 0.4	26 ± 0.2	5 ± 0
21	MC-7 F***	100	83 ± 1	87 ± 0	92 ± 0.5	74 ± 0
22	MC-14 F***	100	99 ± 0	100 ± 0.4	91 ± 0.5	100 ± 0
23	MC-17 F	100	53 ± 1	28 ± 0	0 ± 0	7 ± 0
24	MC-21 F	100	0 ± 0	8 ± 0.3	11 ± 0.3	17 ± 0
25	MC-22 F***	100	84 ± 1	75 ± 0.3	88 ± 0.4	80 ± 0.2
26	MC-23 F	100	0 ± 0	9 ± 0.2	0 ± 0	33 ± 0.4
27	MC-25 F	100	45 ± 1	90 ± 0.2	77 ± 0	81 ± 0.6
28	MC-26 F	100	94 ± 0	87 ± 0.5	97 ± 0.5	91 ± 0.2
	Paclitaxel	1 μM	79 ± 1			
	5-Fluorouracil	20 μM		45 ± 0		
	Adriamycin	1 μM			46 ± 0.7	
	Mitomycin	1 μM				63 ± 0

*** Highly Significant at *P* < 0.05

Out of twenty eight extracts, the extracts showing highest percentage of growth inhibition for all the tested human cancer cell lines were assayed at four different concentrations to check the IC_50_ values (Table 5). Seven extracts were found to be active at least against one of the tested cell lines with IC_50_ of <20 μg mL^−1^. Six extracts showed anticancer activity against A-549 and MCF-7 cell lines each, whereas only five and four extracts were active against HCT-116, and PC-3 cell lines respectively with IC_50_ < 20 μg mL^−1^.

**Table 5:** Anticancer activity (IC_50_ μg mL^−1^) of the extracts prepared from endophytic fungi isolated from *M. citriodora* against different human cancer cell lines

	Tissue	Colon	Lung	Prostate	Breast
S.NO.	Cell line type	HCT-116	A549	PC-3	T47D
	Code	IC_50_ (μg mL^−1^)			
1.	MC-8 L	38.0 ± 0.08^a^	24.3 ± 0.03	26.4 ± 0.06	19.7 ± 0.1^a^
2.	MC-14 L	<10 ± 0.0	<10 ± 0.0	<10 ± 0.0	<10 ± 0.0
3.	MC-18 L	9.0 ± 0.02^a^	4.8 ± 0.02^b^	27.7 ± 0.06^a,b^	19.6 ± 0.02
4.	MC-24 L	<10 ± 0.0	<10 ± 0.0	<10 ± 0.0	<10 ± 0.0
5.	MC-25 L	<10 ± 0.0	<10 ± 0.0	9.9 ± 0.02	12.7 ± 0.02
6.	MC-7 F	32.2 ± 0.2^a^	23.6 ± 0.02^a,b^	32.3 ± 0.04^b^	26 ± 0.04
7.	MC-14 F	62.8 ± 0.5^a,b^	<10 ± 0.0^a,c^	23.3 ± 0.02^b^	42.7 ± 0.3^c^
8.	MC-26 F	11.1 ± 0.01	<10 ± 0.0	11.7 ± 0.01	8.0 ± 0.02
	Paclitaxel in nM	120 ± 0.2	<10 ± 0.0	65 ± 0.3	777 ± 0.1

same letters as superscript depicts significant difference at *P* < 0.05 between IC_50_ values of two cell lines (Additional file 1: Figure S2)

With IC_50_ < 10 μg mL^−1^, six extracts showed cytotoxic activity against one of the tested cell lines, whereas two extracts MC-14 L (*Fusarium oxysporum*), MC-24 L (*Cladosporium tenuissimum*) were active against all the tested cell lines. With same IC_50_ value, four extracts were recorded cytotoxic against HCT-116 cell line, six against A-549 and three against PC-3 and MCF-7 cell lines.

### Antimicrobial activity

The antimicrobial potential of the isolated endophytes was investigated against some human pathogens by agar disc-diffusion and tube dilution method. Crude DCM extracts containing metabolites of these endophytes inhibited the growth of pathogens (Table 6). About 75% of extracts showed antimicrobial activity against tested pathogens with a zone of inhibition (ZI) ranging from 6–35 mm*.* Six (21%), 14 (50%), 7 (25%), 11 (39%), 10 (36%), and 9 (32%) extracts presented significant antimicrobial activity against *E. coli, K. pneumoniae, S. aureus, P. aeruginosa, S. typhimurium,* and *B. subtilis* respectively. Extracts of MC-14 L (*Fusarium oxysporum*) and MC-18 L (*Aspergillus fumigatus*) displayed ZI (9–35 mm) against all the tested human pathogens including *C. albicans*. Extract of MC-18 L (*Aspergillus fumigatus*) displayed better ZI (24–35 mm) than the extract of MC-14 L (*Fusarium oxysporum*) against bacterial pathogens. Extracts of MC-2 L (*Fusarium chlamydosporum*) and MC-24 L (*Cladosporium tenuissimum*) showed antimicrobial activity against all the tested bacterial strains with a ZI 10–26 mm.

**Table 6:** Zone of inhibition (mm) of the extracts prepared from endophytic fungi isolated from *M. citriodora* against different human pathogens

Endophyte	*K. pneumoniae*	*S .aureus*	*E. coli*	*P. aeruginosa*	*S. typhimurium*	*B. subtilis*	*C. albicans*
MC-1 L	-	-	-	-	-	-	-
MC-2 L***	10 ± 0.2	21 ± 0.2	22 ± 0.3	21 ± 0	22 ± 0.2	23 ± 0.2	-
MC-3 L	-	-	-	-	-	-	-
MC-4 L	-	12 ± 0.6	14 ± 0.5	12 ± 0.2	15 ± 0	10 ± 0.2	6 ± 0.4
MC-5 L	10 ± 0.3	-	-	-	-	-	-
MC-6 L	-	-	-	-	-	-	-
MC-8 L	-	9 ± 0.5	-	10 ± 0.3	-	-	-
MC-9 L	-	-	-	25 ± 0.5	-	-	-
MC-10 L	-	15 ± 0.3	-	-	10 ± 0	-	-
MC-12 L	15 ± 0.3	-	14 ± 0.2	12 ± 0.3	16 ± 0.3	-	27.5 ± 0.2
MC-14 L	13 ± 0.5	12 ± 0.2	13 ± 0.6	12 ± 0	15 ± 0.3	11 ± 0.5	11 ± 0.2
MC-15 L	-	14 ± 0.5	-	-	-	-	-
MC-16 L	-	17 ± 0	30	-	12 ± 0.2	18 ± 0.5	10 ± 0
MC-17 L	-	-	-	-	-	-	10 ± 0
MC-18 L***	35 ± 1	24 ± 0	35 ± 0.3	28 ± 0.5	32 ± 0.2	27 ± 0.3	9 ± 1
MC-20 L	-	-	-	-	-	-	-
MC-24 L	10 ± 0.2	22 ± 1	17 ± 0.2	26 ± 0.5	17 ± 0.4	15 ± 0.3	-
MC-25 L	-	-	-	-	-	10 ± 0.4	-
MC-13R	-	10 ± 0.2	-	-	-	10 ± 0.7	-
MC-20R	-	10 ± 0.3	-	10 ± 0.4	-	-	-
MC-7 F	-	-	-	12 ± 0.4	-	-	-
MC-14 F	-	12 ± 0.4	-	10 ± 0.3	-	-	9 ± 1
MC-17 F	-	15 ± 0.4	-	-	-	-	-
MC-21 F	-	-	-	-	-	-	-
MC-22 F	-	-	-	-	17 ± 0.4	13 ± 0.2	-
MC-23 F	-	16 ± 0	22 ± 0.3	-	12 ± 0.5	-	-
MC-25 F	-	-	-	-	-	-	-
MC-26 F	-	-	-	-	-	-	-
Streptomycin	22.5 ± 0.4	18 ± 0.5	18.7 ± 0	25 ± 0.2	20 ± 0.5	18 ± 0.7	-
Amphotericin B	-	-	-	-	-	-	24.6 ± 0.2

*** Highly Significant at *P* < 0.05

The antimicrobial activity of the isolated endophytes was also evaluated by tube dilution method (Table 7). Only eight extracts (27%) displayed antimicrobial potential with the MIC value ranging from 10–100 μg mL^−1^. In the present study, 24.13%, 13.8% and 10.3% extracts showed antimicrobial activity against *S. aureus, E. coli,* and *C. albicans* respectively. Out of eight active extracts, four were found to be active against *S. typhimurium.* Extracts of MC-14 L (*Fusarium oxysporum*), and MC-24 L (*Cladosporium tenuissimum*) showed more potent antimicrobial activity than streptomycin, whereas MC-18 L (*Aspergillus fumigatus*) and streptomycin showed equal antimicrobial potency against *S. typhimurium.* Similarly, Extracts of MC-14 L (*Fusarium oxysporum*), MC-14 F (*Fusarium oxysporum*) and streptomycin were found to be as active against *S. aureus.* MC-14 L (*Fusarium oxysporum*) extract inhibited the growth of all the pathogens used in the study (MIC/MBC 10–25 μg mL^−1^) and was as active as streptomycin against *E. coli*. It inhibited *K. pneumoniae,* and *B. subtilis* with an MIC of 12.5 μg mL^−1^. Extracts of MC-18 L (*Aspergillus fumigatus*) and MC-24 L (*Cladosporium tenuissimum*) showed a broad range of antibacterial activity as it inhibited the growth of all the bacterial pathogens used in the study (MIC/MBC 10–50 μg mL^−1^). Extracts of MC-24 L (*Cladosporium tenuissimum*) inhibited *K. pneumoniae, S. aureus, E. coli,* and *P. aeruginosa* with an MIC of 12.5 μg mL^−1^. Only three extracts MC-14 L *(Fusarium oxysporum)*, MC-14 F (*Fusarium oxysporum*) and MC-16 L (*Neurospora* sp*.*) showed antimicrobial activity against *C. albicans* with an MIC value 25–100 μg mL^−1^.

**Table 7:** Antimicrobial activity (MIC/MBC values in μg mL^−1^) of the extracts prepared from endophytic fungi isolated from *M. citriodora* against different human pathogens

Endophyte	*K. pneumoniae*	*S .aureus*	*E. coli*	*P. aeruginosa*	*S. typhimurium*	*B. subtilis*	*C. albicans*
MC-1 L	-	-	-	-	-	-	-
MC-2 L	>100	25	100	100	100	>100	-
MC-3 L	-	-	-	-	-	-	-
MC-4 L	-	>100	>100	>100	>100	>100	-
MC-5 L	>100	-	-	-	-	-	-
MC-6 L	-	-	-	-	-	-	-
MC-8 L	-	100	-	100	-	-	-
MC-9 L	-	-	-	>100	-	-	-
MC-10 L	-	>100	-	-	>100	-	-
MC-12 L	>100	-	>100	>100	>100	-	-
MC-14 L	12.5	12.5 (MBC)	12.5 (MBC)	25 (MBC)	12.5 (MBC)	12.5	25
MC-15 L	-	>100	-	-	-	-	-
MC-16 L	-	100	>100	-	>100	>100	100
MC-17 L	-	-	-	-	-	-	-
MC-18 L	50	25	25	25	12.5	12.5	>100
MC-20 L	-	-	-	-	-	-	-
MC-24 L	12.5	12.5	12.5	12.5	12.5 (MBC)	25	-
MC-25 L	-	-	-	-	-	>100	-
MC-13R	-	>100	-	-	-	>100	-
MC-20R	-	>100	-	>100	-	-	-
MC-7 F	-	-	-	>100	-	-	-
MC-14 F	-	12.5 (MBC)	-	25	-	-	25
MC-17 F	-	>100	-	-	-	-	-
MC-21 F	-	-	-	-	-	-	-
MC-22 F	-	-	-	-	100	>100	-
MC-23 F	-	>100	>100	-	>100	-	-
MC-25 F	-	-	-	-	-	-	-
MC-26 F	-	-	--	-	-	-	-
Streptomycin	12.5 (MBC)	12.5 (MBC)	12.5 (MBC	12.5 (MBC)	12.5		-
Amphotericin	-	-	-	-	-	**-**	6.25(MBC)

## Discussion

Aromatic and medicinal plants, producers of ethano-pharmacologically important secondary metabolites and essential oils being used in food preservation and in reducing the dose of antibiotic for the treatment of bacterial infections are the legitimate targets to isolate the endophytic fungi. These fungi are utilized in management of plant diseases. This is the first report on the diversity, phylogeny and bioactive potential of endophytic fungi associated with *M. citriodora,* an aromatic and medicinal plant. All the fungal endophytes recovered in the present study represented the phylum Ascomycota, which is one of the most diverse and ubiquitous phyla of eukaryotes covering approximately 8% of the Earth’s landmasses [40, 41]. The most prevalent class of fungi was Sordariomycetes followed by Eurotiomycetes and Dothideomycetes. The endophytes of *M. citriodora* were compared with the endophytes of *Ocimum sanctum* because they both belong to same family and both are aromatic plants. Endophytes associated with *Ocimum sanctum,* showed the same representation of classes except Saccharomycetes [41]. The difference in the endophytic communities between the *M. citriodora* and *Ocimum sanctum* might be because of host specificity as well as their interactions with different ecological niches.

Further, variation was also observed in the spatial distribution of endophytic community of the *M. citriodora* suggesting that distinct micro-environments of tissues are responsible for shaping their micro-biota differently. Thus, tissue specificity was evident among the endophytes of the *M. citriodora.* Leaves supported the wider diversity of endophytes followed by flowers and roots. This might be because of greater surface area of leaves exposed to outer environment. These results well corroborate with the previous studies of endophytic fungi isolation on various Indian medicinal plants [42, 43].

In present study, dark septate fungi such as *Curvularia aeria* and *Alternaria alternata* were isolated from leaves contrary to their common habitat i.e. root tissue [44, 45].

For searching the safer and novel drug based on natural product, endophytic extracts were screened for cytotoxic and antibacterial activity. Nearly 72% of the extracts showed cytotoxic activity against one of the tested cell lines while 39% of the extracts were active against all the tested cell lines with >50% growth inhibition. IC_50_ < 20 μg mL^−1^ (cytotoxicity criteria given by National Cancer Institute for the screening the crude plant extracts) was shown by 25% of extracts [46]. In comparison, Carvalho et al. [47] have reported that three out of sixteen endophytic extracts isolated from the plant *Stryphnodindron adstringens* (Mart.) Coville (Fabaceae), had IC_50_ 20 μg mL^−1^ against MCF-7 cell line, whereas Zhao et al. [48] ascertained the cytotoxicity of the extract of endophyte *Hypocrea lixii* R18 (IC_50_ = 29.8 μg mL^−1^) against PC-3 cell line. This was found to be less cytotoxic than four extracts [MC-14 L (*Fusarium oxysporum*), MC-24 L (*Cladosporium tenuissimum)*, MC-25 L (*Fusarium* sp*.*), MC-26 F (*Fusarium oxysporum*)] used in the present study against the same cell line.

Overall MC-8 L (*Aspergillus* oryzae), MC-14 L (*Fusarium oxysporum*), MC-18 L (*Aspergillus fumigatus*), MC-24 L (*Cladosporium tenuissimum)*, MC-25 L (*Fusarium* sp*.*), MC-14 F (*Fusarium oxysporum*), MC-26 F (*Fusarium oxysporum*) extracts were found most promising in terms of cytotoxic activity (IC_50_ < 10 μg mL^−1^) against different human cancer cell lines.

The cytotoxic molecules beauvericin (IC_50_ = 1.42 μg mL^−1^) and bikaverin (IC_50_ = 0.161 μg mL^−1^) were isolated from an endophytic *Fusarium oxysporum* [49]. Till now approximately more than 500 compounds have been isolated from *Fusarium* genera. Out of which, 26 compounds have cytotoxic activity. On the basis of natural product dictionary records, only one cytotoxic molecule is known from *Fusarium oxysporum* while no cytotoxic molecule is reported from *Cladosporium tenuissimum*. Thus there is lot of scope of isolating new cytotoxic compounds from these endophytes [MC-14 L (*Fusarium oxysporum*), MC-24 L (*Cladosporium tenuissimum*).

The antimicrobial potential of the endophytes isolated from *M. citriodora* was investigated against human pathogens by agar disc-diffusion and tube dilution methods. About 75% extracts showed antimicrobial activity against the tested pathogens with a zone of inhibition (ZI) ranging from 6-35 mm which is a higher percentage in comparison to 48.3% active endophytic bacterial extracts isolated from *Aloe vera* with the ZI 6–12 mm [50]. This indicates that endophytes from *M. citriodora* have better antimicrobial potential than endophytic bacteria from *Aloe vera.* In present study, 50% and 25%, extracts showed antimicrobial activity against *S. aureus* and *E. coli* respectively. This is in contrast to Guimaraes et al. [51] who screened 39 endophytic fungal extracts and found that 5.1%, 25.6% and 64% extracts to be active against *S. aureus, E. coli* and *C. albicans* respectively. Hazalin et al. [52] screened 300 endophytic extracts against human pathogens. out of which only 8% extracts were active. Wiyakrutta et al. [53] also screened 360 endophytic extracts against *Mycobacterium tuberculosis* and found 90 (25%) extracts to be active. The highest number of active extracts (50%) against *S. aureus* in the present study indicates that endophytic fungi isolated from *M. citriodora* might be used to produce potent antimicrobial compounds.

The antimicrobial potential of the endophytic extracts was evaluated by tube dilution method. Only eight extracts (27%) displayed antimicrobial activity against the tested human pathogens with an MIC value ranging from 10–100 μg mL^−1^. On comparing the two methods, it was found that upon using agar disc-diffusion, twenty one extracts were found to be active, whereas by using tube dilution method only eight extracts were found to be active with MIC value ≤100 μg mL^−1^. Extract of MC-14 L (*Fusarium oxysporum*), which displayed ZI between 11–15 mm, showed better antimicrobial activity with an MIC/MBC value 12.5–25 μg mL^−1^ against all the tested pathogens, whereas MC-18 L (*Aspergillus fumigatus*), which displayed better ZI (24–35 mm), showed little bit lower MIC value (12.5–50 μg mL^−1^). Results suggest that difference in antimicrobial potency may be because a more diffusible but less active extract may give a bigger zone of inhibition than a non-diffusible but more active extract.

In present study, extracts inhibited the human pathogens with an MIC value of 12.5 μg mL^−1^ whereas in another study crude extracts of endophytes *Papulaspora immersa* and *Arthrinium arundinis* isolated from *Smallanthus sonchifolius* inhibited the human pathogens with an MIC value of 90–280 μg mL^−1^ [54]. Akinsanya et al. [50] reported that the MIC and MBC values of the endophytic bacterial extracts isolated from *Aloe vera* range from 0.625 to 20 mg mL^−1^ against some of the bacterial indicators, which is quite low when compared to the present study. In another study, two endophytic extracts were found to be active against *Microsporum gypseum* with an MIC < 12.5 μg mL^−1^ [55].

Present study along with previous studies have indicated that *Fusarium* spp*.* are the most dominant species among endophytes producing bioactive compounds such as pentaketide (CR377: 2-methylbutyraldehyde-substituted-α-pyrone), beauvericin, subglutinol A and B [56–58]. These molecules were found active against *C. albicans,* and methicillin-resistant *S. aureus.* The *Fusarium* spp*.* were isolated from *Selaginella pallescens, Cinnamomum kanehirae, Tripterygium wilfordii* plants respectively [56–58].

In terms of antimicrobial activities extracts of MC-14 L (*Fusarium oxysporum),* MC-18 L (*Aspergillus fumigatus*), MC-24 L (*Cladosporium tenuissimum*) and MC-14 F (*Fusarium oxysporum*) were found promising. Only three antibacterial and 5 antifungal molecules are known from *Fusarium oxysporum* while no antibacterial and antifungal molecule is known from *Cladosporium tenuissimum.* Thus these endophytes [MC-14 L (*Fusarium oxysporum*), MC-24 L (*Cladosporium tenuissimum*) could be a source of new antimicrobial compounds.

## Conclusion

In conclusion, this is the first report of diversity of fungal endophytes isolated from *M. citriodora*. These results indicated that *M. citriodora* harbors a rich fungal endophytic community with anticancer and antimicrobial activities. Extracts of MC-14 L (*Fusarium oxysporum)* and MC-24 L (*Cladosporium tenuissimum*) were found to have potent cytotoxic and antimicrobial properties thus could be a source of novel cytotoxic/antimicrobial compounds highlighting their potential use in the development of drugs (anti-cancer/antimicrobial), but needs to be further investigated at molecular and mechanistic level.

## Additional file

Additional file 1: Morphological and microscopic characterization of endophytes isolated from *Monarda citriodora.* **Table S1.** Morphological Characteristics of endophytes isolated from *Monarda citriodora* L. **Figure S1.** Microscopic images of endophytes isolated from *Monarda citriodora* L.]. The ITS sequences from endophytic isolates are available via the following links: http://www.ncbi.nlm.nih.gov/nuccore/KU527781; KU680345; KU680346. Rest of raw datasets of bioactivity used and/or analysed during the current study can be available from the corresponding author on reasonable request. (DOC 2192 kb)

## Abbreviations

IC_50_: Concentration of extract at which 50% growth inhibition of cell line occurs
MBC: Minimum bactericidal concentrations
MIC: Minimum inhibitory concentration
MTT: 3-(4, 5-dimethylthiazol-2-yl)-2, 5-diphenyltetrazolium bromide
ZI: Zone of inhibition.
