# Supplementary material for: Diversity, Phylogeny, anticancer and antimicrobial potential of fungal endophytes associated with Monarda citriodora L
Source: BMC Microbiol. 2017 Mar 7;17:44. doi: 10.1186/s12866-017-0961-2 (PMC5339955; doi:10.1186/s12866-017-0961-2)
Supplement: Additional file 1: — Morphological and microscopic characterization of endophytes isolated from Monarda citriodora. Table S1. Morphological Characteristics of endophytes isolated from Monarda citriodora L. Figure S1. Microscopic images of endophytes isolated from Monarda citriodora L.]. The ITS sequences from endophytic isolates are available via the following links: http://www.ncbi.nlm.nih.gov/nuccore/KU527781; KU680345; KU680346. Rest of raw datasets of bioactivity used and/or analysed during the current study can be available from the corresponding author on reasonable request. (DOC 2192 kb) [file 12866_2017_961_MOESM1_ESM.doc]

**Morphological and microscopic characterization of endophytes isolated from *Monarda citriodora* L.**

**Table S1: Morphological Characteristics of endophytes isolated from *Monarda citriodora* L.**

| **Endophyte** | **colour** | **Morphology** |
| --- | --- | --- |
| MC-1L | orangish | Orange filaments with black spores |
| MC-2L | brownish | highly filamentous |
| MC-3L | dark black | filamentous |
| MC-4L | Dark green | Highly sporogenous |
| MC-5L | black | filamentous |
| MC-6L | green and white | alternate patches of green and white |
| MC-7F | white and pink | highly filamentous |
| MC-8L | Parrot green | highly sporogenous |
| MC-9L | green (in patches) | highly sporogenous |
| MC-10L | White | filamentous |
| MC-11L | Blackish | highly sporogenous |
| MC-12L | Orangish brown | velvety |
| MC-13R | Grey with orange background | Velvety and filamentous |
| MC-14L | Oranges with brown background | slightly filamentous |
| MC-14F | pink | filamentous |
| MC-15L | Orangish brown | filamentous |
| MC-16L | white | Sticky and velvety |
| MC-17L | sea green | velvety |
| MC-17F | Black | Sporogenous |
| MC-18L | green | Highly sporogenous |
| MC-20L | Dark green | Filamentous |
| MC-20R | Highly blackish green | Sporogenous |
| MC-21F | Dark green | Sporogenous |
| MC-22F | Pink and purple | Filamentous |
| MC-23F | Pink and purple concentric rings | Filamentous |
| MC-24L | Green and orange | Highly sporogenous |
| MC-25F | Purple and white | Filamentous |
| MC-25L | Pink and white | Filamentous |
| MC-26F | pink | Filamentous |

**Figure S1: Microscopic images of endophytes of endophytes isolated from *Monarda citriodora* L.**


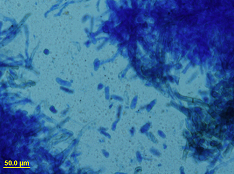

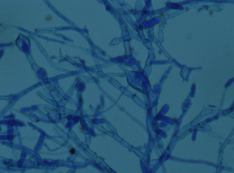

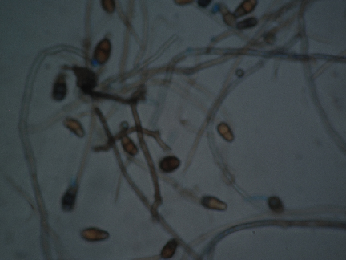


MC-1-L

MC-2-L

MC-3-L


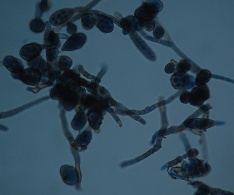

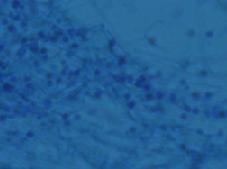

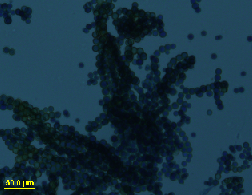


MC-4-L

MC-6-L

MC-8-L


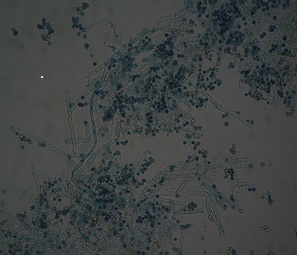

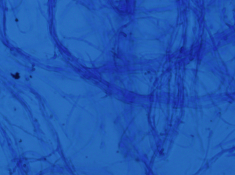

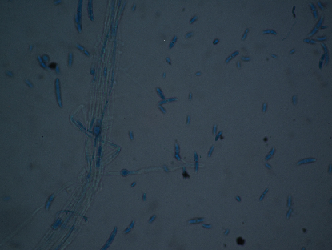


MC-9-L

MC-10-L

MC-12-L


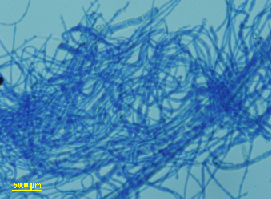

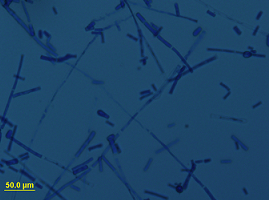

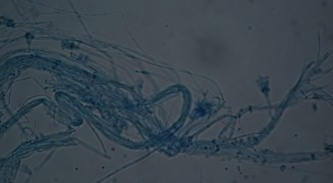


MC-15-L

MC-16-L

MC-17-L


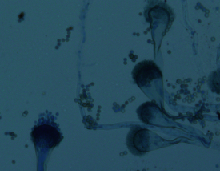

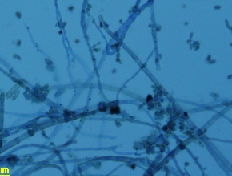

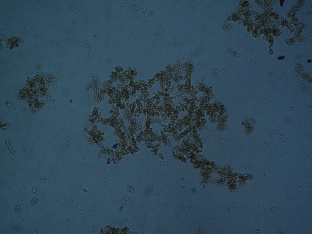


MC-18-L

MC-20-L

MC-24-L


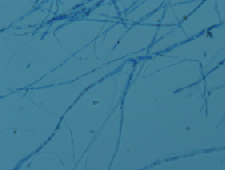

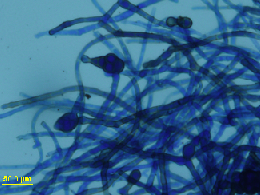

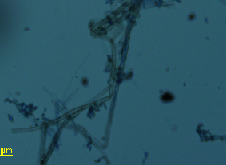


MC-20-R

MC-25-L

MC-13-R


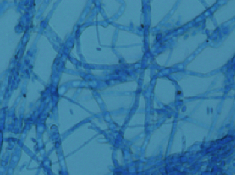

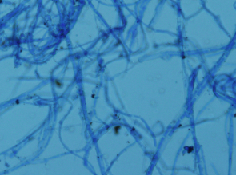


MC-7-F

MC-17-F

**Figure S2: Comparative cytotoxic activity of endophytes isolated from *Monarda citriodora* L. against HCT-116, A-549, MCF-7, PC-3 cell lines generated after ANOVA and TUKEYS post hoc analysis**
